# Depression in Patients on Hemodialysis: A Dilapidated Facet

**DOI:** 10.7759/cureus.29077

**Published:** 2022-09-12

**Authors:** Bhaskaran Shanmukham, Mahendra Varman, Sarojini Subbarayan, Varatharajan Sakthivadivel, Ariyanachi Kaliappan, Archana Gaur, Lakshmi Jyothi

**Affiliations:** 1 General Medicine, Melamruvathur Adhiparasakthi Institute of Medical Sciences and Research, Melmaruvathur, IND; 2 Pediatrics, Sri Muthukumaran Medical College Hospital and Research Institute, Chennai, IND; 3 General Medicine, All India Institute of Medical Sciences - Bibinagar, Hyderabad, IND; 4 Anatomy, All India Institute of Medical Sciences - Bibinagar, Hyderabad, IND; 5 Physiology, All India Institute of Medical Sciences - Bibinagar, Hyderabad, IND; 6 Microbiology, All India Institute of Medical Sciences - Bibinagar, Hyderabad, IND

**Keywords:** hypertension, bdi score, hemodialysis, esrd, depression

## Abstract

Introduction

End-stage renal disease (ESRD) has increased in India due to the growing load of non-communicable diseases. The most prevalent psychological issue among these patients has been identified as depression, which may have an impact on treatment success. Around 20% to 90% of hemodialysis patients experience depression. The current study aimed to determine the prevalence of depression among patients undergoing hemodialysis as well as the relationship between this condition and the sociodemographic and clinical parameters of the patients.

Methods

Basic demographic information and particulars of chronic morbidity, duration, and the number of cycles of hemodialysis per week were noted. The Beck Depression Inventory (BDI) score was administered to screen for depression.

Results

A total of 92 participants were enrolled in the study; 69 (75%) were males. The mean age of participants was 52 years. Hypertension (100%) was the most common co-morbidity followed by diabetes mellitus (38%). The mean duration of chronic kidney disease was 3.9 years. The majority (68.5%) had hemodialysis twice per week. Forty-one percent (41%) screened positive for borderline clinical depression or more. The mean BDI score was 17.07. The number of hemodialyses per week had a significant relation with depression with an odds ratio of 4.16 and 95% CI of 1.4-12.38.

Conclusion

Depression is prevalent among patients with chronic kidney disease who are on dialysis. The management of this preventable illness demands a repertoire of measures such as launching a program for the detection and treatment of depression combining psychiatric professionals and social volunteer organizations.

## Introduction

End-stage renal disease (ESRD) has increased in India due to the growing load of non-communicable diseases. It is established that diabetes, hypertension, and obesity can worsen the age-related decline in renal function linked to chronic kidney disease (CKD). Based on the estimated glomerular filtration rate (eGFR), CKD may be divided into five stages. GFR of < 60 mL/min/1.73 m2 is abnormal in all age groups. The most severe type of CKD is an end-stage renal disease, also known as stage V chronic kidney disease (CKD V), which occurs when the kidneys cannot successfully maintain homeostasis [1]. The prevalence of end-stage renal disease is likely to be between 0.79% and 1.4% in India [2]. In India, about 2.2 lakh new patients with CKD V are added every year, increasing the demand for dialysis [3]. The main goal of hemodialysis is to re-create the intracellular and extracellular fluid environment essential for a healthy life. This is done by removing waste products and transferring solutes from the dialysate, including bicarbonates, into the blood. Hemodialysis makes the patient completely dependent on a machine and medical staff. In addition, the patient needs to take various medications and adhere to a strict diet. The high expense of the therapy and loss of working days result in a situation where the patient is subjected to a severe financial burden. All these ultimately result in a deleterious effect on the mental health of the patients receiving hemodialysis [4].

The patient's daily routine is severely disrupted in many ways including the selection of food and fluids for daily intake and lifelong dependence on hemodialysis, medical professionals, and family members. The most prevalent psychological issue among CKD patients has been identified as depression, which may have an impact on treatment success. Around 20% to 90% of hemodialysis patients experience depression [5]. Although most physicians know this, they frequently lack time to properly examine and deal with patients' emotional situations. The current study sought to determine the prevalence of depression among patients undergoing hemodialysis as well as the relationship between this condition and the clinical parameters of the patients.

## Materials and methods

This cross-sectional study was conducted in the dialysis clinic of a tertiary care hospital situated in the northern part of Tamilnadu after obtaining ethical approval from the Institutional Ethics Committee (Human Studies) (MAPIMS/IEC/52/2022). An earlier study estimated that 45% of the participants would have mild to severe depression. For a required precision of 10% and a 95% confidence level, the minimum required sample size was 95 [6]. A consecutive sampling technique was used to achieve the sample size.

On average, our dialysis unit receives 40 patients per day. The particulars of each patient were maintained in a register. Patients who attended the dialysis clinic for maintenance hemodialysis between May and July 2022 were included in the study after obtaining written informed consent. Patients who were already on anti-depressants and those who were not willing to participate were excluded from the study. A semi-structured interview schedule was used to collect basic demographic information and particulars of chronic morbidity. This was followed by the administration of the Beck Depression Inventory (BDI) version II to screen for depression. It is a self-reported rating inventory comprising 21 items that measure characteristic attitudes and symptoms of depression [7]. The questionnaire was translated to the local language, back-translated into English, and suitably modified (see Appendices). This was pre-tested and modified before use. Patients who had depression as per BDI scoring were referred to a psychiatrist for consultation and management.

Statistical analysis

Continuous variables were represented as mean±standard deviation. Categorical variables were represented as frequency and percentage. An unpaired t-test was used to compare continuous variables between the groups. Chi-square was used to analyze the categorical variables between the groups. Multi-variable logistic regression analysis was done to estimate the probability of depression with variable risk factors. P-value <0.05 was considered statistically significant.

## Results

A total of 100 participants were enrolled in the study. Eight participants were excluded due to incomplete data. The final sample size was 92. Of this, 69 (75%) were males, and the remaining were females. The mean age of participants was 52 years. All the participants had hypertension, either primary or secondary in origin, following chronic kidney disease. Among the participants, 38% had diabetes as a comorbidity, and 4.3% had thyroid-related problems. The mean duration of chronic kidney disease (CKD V) was 3.88 years. The majority (68.5%) had hemodialysis twice per week, and 14.1 were admitted to the ICU during the study period. The mean BDI score was 17.07 (Table 1).

**Table 1:** Characteristics of the study population CKD: chronic kidney disease; BDI: Beck Depression Inventory

Parameter	N=92 Frequency (percentage)
Mean age in years (SD)	52.02±10.98
Gender	
Male	69 (75)
Female	23 (25)
Hypertension	92 (100)
Diabetes mellitus	35 (38)
Mean duration of diabetes mellitus in years (SD)	3.91 (7.0)
Thyroid disorder	4 (4.3)
Myocardial infarction	1 (1.1)
Mean duration of CKD in years (SD)	3.88 (2.9)
Number of Hemodialyses twice per week	63 (68.5)
Number of Hemodialyses thrice per week	29 (31.5)
Mean duration of Hemodialysis in years (SD)	3.68 (2.9)
ICU admission	13 (14.1)
Hospital admission	69 (75)
Mean BDI score (SD)	17.07 (12.7)

**Figure 1:**
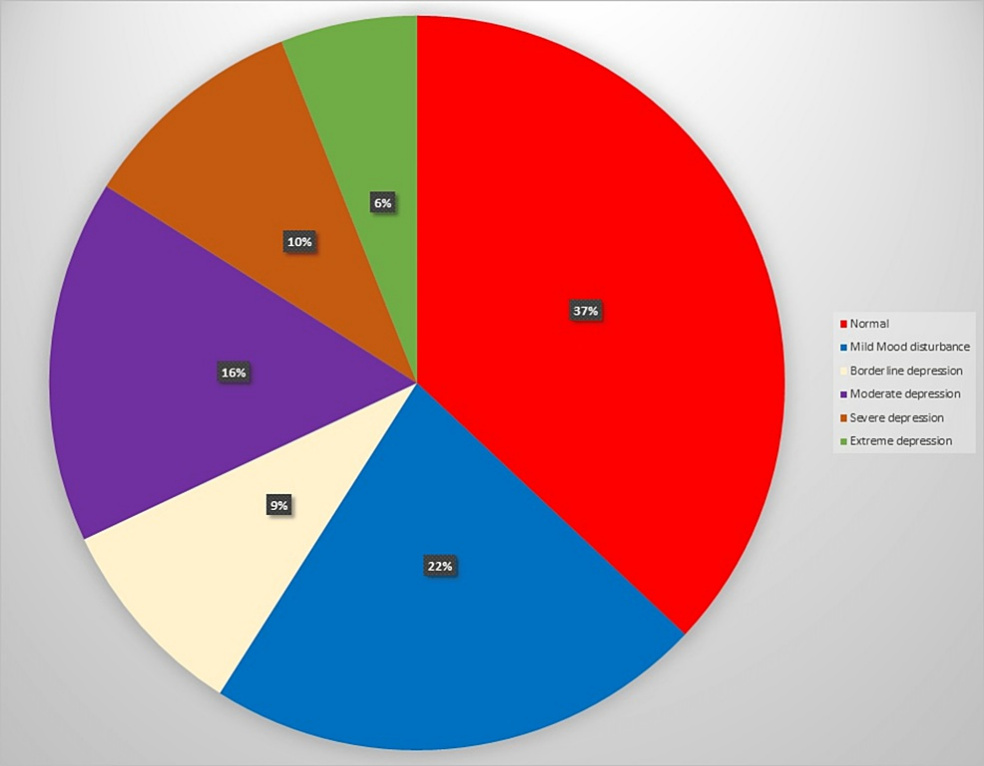
Categorization of participants based on the Beck Depression Inventory

It was observed that 41% screened positive for borderline clinical depression or more, and only 37% were detected as normal (Figure 1).

In order to find out the association between selected demographic and clinical variables with depression, those with borderline clinical depression or more were considered depressed, and various clinical variables were compared with these categories. It was observed that depression was higher among males, those aged less than 50 years, non-diabetics, and a smaller duration of chronic kidney disease, hypertension, and hemodialysis; but these results were not statistically significant. Those who had hemodialysis two times a week had significant depression (Table 2).

**Table 2:** Relation between demographic, clinical variables, and presence of depression CKD: chronic kidney disease, * represents P-value <0.05

Parameter	Depression (Yes) n=38	Depression (No) n=54	P value
Mean age in years (SD)	50.11 (11.8)	53.37 (10.2)	0.173
Gender			0.311
Male	27 (71.1)	42 (77.8)	
Female	11 (28.9)	12 (22.2)	
Mean duration of hypertension in years (SD)	5.13 (4.15)	6.94 (6.53)	0.118
Diabetes mellitus	12 (31.6)	23 (42.6)	0.197
Mean duration of diabetes mellitus in years (SD)	3.11 (6.2)	4.48 (7.5)	0.282
Thyroid disorder	-	4 (7.4)	0.113
Myocardial infarction	-	1 (1.9)	0.587
Mean duration of CKD in years (SD)	3.45 (2.4)	4.19 (3.2)	0.340
Number of Hemodialyses per week			0.055*
Twice per week	22 (57.9)	41 (75.9)	
Thrice per week	16 (42.1)	13 (24.1)	
Mean duration of Hemodialysis in years (SD)	3.29 (2.5)	3.96 (2.9)	0.340
ICU admission	5 (13.2)	8 (14.8)	0.537
Hospital admission	26 (68.4)	43 (79.6)	0.164

On multivariable logistic regression, the number of hemodialysis per week had a significant relationship with depression, with an odds ratio of 4.16 and 95% CI of 1.4-12.38 (Table 3).

**Table 3:** Multivariable logistic regression for prediction of depression CKD: chronic kidney disease; RR: relative risk; CI: confidence interval; * represents P-value <0.05

Parameter	RR (95% CI)	P value
Age	0.96 (0.91-1.01)	0.151
Gender	1.89 (0.6-5.98)	0.273
Duration of hypertension	0.99 (0.86-1.15)	0.952
Diabetes mellitus	1.46 (0.29-7.16)	0.640
Duration of diabetes mellitus	0.99 (0.86-1.14)	0.964
Thyroid disorder	-	0.999
Myocardial infarction	-	1.000
Duration of CKD	0.93 (0.72-1.19)	0.575
Number of Hemodialyses per week	4.16 (1.4-12.38)	0.010*
Duration of Hemodialysis	0.84 (0.64-1.11)	0.234
ICU admission	1.09 (0.26-4.51)	0.900
Hospital admission	1.89 (0.63-5.7)	0.253

## Discussion

CKD constitutes one of the most incapacitating illnesses in the world, with an 8-16% global prevalence in 2013 and an 11-13% global prevalence in 2016 [8]. Psychiatric problems commonly co-exist with most chronic diseases, including CKD. Numerous studies support the significant frequency of depression in CKD patients. Depression is thought to affect 23.7% of CKD patients. Furthermore, compared to individuals not receiving dialysis, CKD patients receiving dialysis are 34.5% more likely to have depression [9]. In this study on 92 patients in a dialysis clinic using the Beck Depression Inventory, we observed that around 41% of the dialysis patients screened positive for depression. According to a study by Chilcot et al., the Beck Depression Inventory is an effective instrument for detecting depression [10]. A systematic review by Gregg et al. reveals that BDI has been used widely for screening depression though the cut-off for the same has varied from a score of 11 to 20 and above to screen for depression [11]. In our study though, the cut-off was 17 and above. Ahlawat et al., in their study, reported that the prevalence of depression in CKD patients is 44.7% [6]. In a similar study by Rai et al., in a state-run dialysis clinic in New Delhi using the same tool, 47.8% were observed to have depressive symptoms [12]. A study on 47 hemodialysis patients done in Delhi by Joshua et al. revealed that 72.3% had depression, however, the cut-off of 11 and above was used for the BDI [13].

In this study, it was observed that, among the CKD patients with depression, 71.1% are males and 28.9% are females, though these values are not found to be statistically significant. In a study by Mosleh et al., among the CKD patients on dialysis, female gender and old age were significantly associated with depression [14]. However, another study done among CKD patients in Pakistan showed that men had more depression and anxiety than women [15]. Another study, however, found no gender differences in the depressive symptoms of hemodialysis patients [16]. Regarding the sex difference in our study, it could be postulated that considering cultural barriers, men may exhibit more anxiety and depression when under financial stress; they become more reliant on other family members rather than fulfilling their social obligations. In this study, most patients with depression are around 50 years of age. This is in line with the findings of other studies where older patients presented a lower level of physical well-being with a higher degree of depression [17-19]. This could be attributed to older patients reportedly losing interest in social pursuits, becoming more socially isolated, and experiencing depression.

In the present study, most patients with depression have associated diabetes for a mean duration of three years and hypertension for around five years, though they are statistically insignificant. This contrasts with a previous study that showed a strong association between disease comorbidities and depression in CKD [20]. A similar study also found an association between depression in CKD and comorbidities and suggested that depression can lead to the exacerbation of comorbid conditions [21]. Patients with CKD are known to experience depression at higher rates when they have diabetes. Depression is linked to higher health care use, worse treatment compliance, and subpar social and vocational performance in people with CKD and associated diabetes [22-24]. Another study reported that significant depression at baseline was associated with a 2.95-fold greater risk of mortality among CKD V diabetic patients [25]. In the present study, 26% of hospitalized CKD patients developed depression though it is not statistically significant. The previous study has shown that the proportion of CKD hospitalizations with depression doubled from 2005 to 2013 (5.01-11.78%) [26]. Patients who were depressed had prolonged hospitalization. Previous studies have shown that rates of depression are higher in long-term care facilities, and it is possible that more patients with depression were coming from these facilities [27].

In the present study, logistic regression showed that the number of hemodialysis per week is significantly associated with the development of depression. According to a prior study, depression and anxiety in hemodialysis patients may progress in diverse ways during dialysis [28]. Another study showed no significant difference between the duration of hemodialysis and depression [14]. In another study, though the standard duration of hemodialysis sessions is more than 3.5 hours per session in patients with depression, no significant difference was found between patients who had hemodialysis sessions of less than 3.5 hours or who had hemodialysis sessions of 3.5 hours and more and the depression score level [20]. According to a qualitative study, people with CKD may suffer detrimental psychological consequences from prolonged dialysis [29]. Another study also showed that the number of hours of hemodialysis is significantly associated with depression [30]. This could be attributed to the patient's existing health state and awareness of their diminished control over their health and invasive hemodialysis operations such as placing a needle into the arteriovenous fistula, implanting central venous catheters, hearing alarms from the dialysis machine, and having the renal staff switch shifts at the dialysis station. 

Limitations

Our study was done in a single center with a minimal sample size, which limits the external validity. Large-scale multicentric studies may be planned to validate our findings in various ethnic populations.

## Conclusions

Depression is prevalent among patients with chronic kidney disease who are on dialysis; it is associated with poor quality of life. Patients generally deny depressive illness for the fear of stigmatization. Depression can lead to discontinuation of treatment including hemodialysis and may progress to suicidal tendencies.

In our study, age, gender, co-morbidities, duration of chronic kidney disease, and hospitalization were not associated with depression. The number of hemodialyses per week was significantly associated with depression. Different approaches should be taken into consideration for identifying depressed CKD patients and enhancing the healthcare system for these patients. These include starting a program for identifying and treating depression by involving psychiatric experts and supportive social volunteer groups.

## Appendices

The Beck Depression Inventory

1.

0 I do not feel sad.

1 I feel sad

2 I am sad all the time and I can't snap out of it.

3 I am so sad and unhappy that I can't stand it.

2.

0 I am not particularly discouraged about the future.

1 I feel discouraged about the future.

2 I feel I have nothing to look forward to.

3 I feel the future is hopeless and that things cannot improve.

3.

0 I do not feel like a failure.

1 I feel I have failed more than the average person.

2 As I look back on my life, all I can see is a lot of failures.

3 I feel I am a complete failure as a person.

4.

0 I get as much satisfaction out of things as I used to.

1 I don't enjoy things the way I used to.

2 I don't get real satisfaction out of anything anymore.

3 I am dissatisfied or bored with everything.

5.

0 I don't feel particularly guilty

1 I feel guilty a good part of the time.

2 I feel quite guilty most of the time.

3 I feel guilty all of the time.

6.

0 I don't feel I am being punished.

1 I feel I may be punished.

2 I expect to be punished.

3 I feel I am being punished.

7.

0 I don't feel disappointed in myself.

1 I am disappointed in myself.

2 I am disgusted with myself.

3 I hate myself.

8.

0 I don't feel I am any worse than anybody else.

1 I am critical of myself for my weaknesses or mistakes.

2 I blame myself all the time for my faults.

3 I blame myself for everything bad that happens.

9.

0 I don't have any thoughts of killing myself.

1 I have thoughts of killing myself, but I would not carry them out.

2 I would like to kill myself.

3 I would kill myself if I had the chance.

10.

0 I don't cry any more than usual.

1 I cry more now than I used to.

2 I cry all the time now.

3 I used to be able to cry, but now I can't cry even though I want to.

11.

0 I am no more irritated by things than I ever was.

1 I am slightly more irritated now than usual.

2 I am quite annoyed or irritated a good deal of the time.

3 I feel irritated all the time.

12.

0 I have not lost interest in other people.

1 I am less interested in other people than I used to be.

2 I have lost most of my interest in other people.

3 I have lost all of my interest in other people.

13.

0 I make decisions about as well as I ever could.

1 I put off making decisions more than I used to.

2 I have greater difficulty in making decisions more than I used to.

3 I can't make decisions at all anymore.

14.

0 I don't feel that I look any worse than I used to.

1 I am worried that I am looking old or unattractive.

2 I feel there are permanent changes in my appearance that make me look unattractive

3 I believe that I look ugly.

15.

0 I can work about as well as before.

1 It takes an extra effort to get started at doing something.

2 I have to push myself very hard to do anything.

3 I can't do any work at all.

16.

0 I can sleep as well as usual.

1 I don't sleep as well as I used to.

2 I wake up 1-2 hours earlier than usual and find it hard to get back to sleep.

3 I wake up several hours earlier than I used to and cannot get back to sleep.

17.

0 I don't get more tired than usual.

1 I get tired more easily than I used to.

2 I get tired from doing almost anything.

3 I am too tired to do anything.

18.

0 My appetite is no worse than usual.

1 My appetite is not as good as it used to be.

2 My appetite is much worse now.

3 I have no appetite at all anymore.

19.

0 I haven't lost much weight, if any, lately.

1 I have lost more than five pounds.

2 I have lost more than ten pounds.

3 I have lost more than fifteen pounds.

20.

0 I am no more worried about my health than usual.

1 I am worried about physical problems like aches, pains, upset stomach, or constipation.

2 I am very worried about physical problems and it's hard to think of much else.

3 I am so worried about my physical problems that I cannot think of anything else.

21.

0 I have not noticed any recent change in my interest in sex.

1 I am less interested in sex than I used to be.

2 I have almost no interest in sex.

3 I have lost interest in sex completely.

 Total Score____________________Levels of Depression

1-10____________________These ups and downs are considered normal

11-16___________________ Mild mood disturbance

17-20___________________Borderline clinical depression

21-30___________________Moderate depression

31-40___________________Severe depression

over 40__________________Extreme depression
